# Is qPCR always the most sensitive method for malaria diagnostic quality surveillance?

**DOI:** 10.1186/s12936-023-04822-w

**Published:** 2023-12-15

**Authors:** Cristian Koepfli

**Affiliations:** Department of Biological Sciences and Eck Institute for Global Health, University of Notre Dame, Notre Dame, USA

## Abstract

In many studies to evaluate the quality of malaria diagnosis, microscopy or rapid diagnostic tests (RDT) are compared to PCR. Depending on the method for sample collection and storage (whole blood or dried blood spot), volume of blood used for extraction, volume of DNA used as PCR template, and choice of PCR target (single vs. multi-copy gene), the limit of detection (LOD) of PCR might not exceed the LOD of expert microscopy or RDT. One should not assume that PCR always detects the highest number of infections.

## Background and discussion

In a recent study, Abebe and colleagues found a large number of patients diagnosed to carry *Plasmodium falciparum*/*Plasmodium vivax* mixed infections by microscopy or rapid diagnostic test (RDT) to be mono-infections by qPCR [1]. Among febrile patients in Ethiopia, only 20/34 mixed infection detected by RDT, and 18/68 mixed infection detected by expert microscopy were confirmed by qPCR [1]. The authors propose that RDT and expert microscopy yielded incorrect results, and called for a strengthening of routine malaria diagnostic methods. One needs, however, to question whether qPCR could have missed mixed infections.

The limit of detection (LOD) of molecular diagnostics is impacted by the type of input sample used, e.g., dried blood spot (DBS) or whole blood, blood volume analysed, DNA extraction protocol, and qPCR assay. The authors have provided the details needed to calculate a rough estimate of the LOD of their assay. They extracted DNA from one 6 mm-punch of DBS using the QIAamp DNA extraction kit and eluted the DNA into a volume of 100 µL. One 6 mm punch of DBS is expected to harbour the equivalent of approximately 15 µL blood [2], thus the DNA was diluted approximately sevenfold during extraction (from 15 to 100 µL). Holzschuh and Koepfli previously showed that only approximately 10% of DNA is recovered from a DBS during the lysis step, and that the QIAamp kit recovers approximately 25% of DNA [3]. Considering dilution and loss of DNA during extraction, the number of genomes per µL extracted DNA was reduced by a factor of 280 compared to initial parasite density (sevenfold dilution * tenfold reduction of DNA during lysis of DBS * fourfold reduction during DNA clean up). The study authors used 5 µL of extracted DNA as template for qPCR and amplified a mitochondrial gene [4], which is expected to be present in around 10 copies per parasite [5]. Let’s assume an infection with an initial density of 10 parasites/µL, resulting in 100 mitochondrial genomes per µL blood. After extraction, 0.35 (100/280) mitochondrial genomes are expected per µL DNA. The 5 µL DNA used as template correspond to less than 2 mitochondrial genomes (5 * 0.35) added to the qPCR. It is difficult to determine the minimal number of templates that a PCR is able to amplify, and thus whether the sample in this example would reliably result in a positive PCR result. Many replicates with well-quantified template densities around the limit of amplification would need to be run to determine that. While some PCRs are able to yield a positive result from as little as one template, others require higher template densities.

Even with the actual efficacy of DNA extraction of the current study likely to differ from published estimates due to small differences in the protocols, it is possible that the LOD of the qPCR was well above 10 parasites/µL blood. Both expert microscopy [6, 7] and *P. falciparum* RDTs [8, 9] might detect parasites at densities as low as 10–50 parasites/µL. Expert microscopy and RDT might thus reach similar, or better LOD than qPCR (of note, Abebe and colleagues used the SD Bioline Malaria Ag Pf/Pv RDT, which is less sensitive than the latest generation of RDTs).

Challenges in diagnosis are even more pronounced among asymptomatic infections that are typically of low density [10]. Co-infections of *P. falciparum* and *P. vivax* might result in modulation of parasite densities [11, 12], thus adding to the discrepancies in the detection of co-infections by different diagnostic tools by Abebe and colleagues. When comparing microscopy and PCR to RDT data, further complexities arise as the concentration of antigens detected by RDTs do not always correlate with parasite densities [9]. Antigens can persist after parasite clearance [13], which could also explain some of the mixed infection detected by RDT where only one species was detected by PCR.

In summary, Abebe and colleagues highlighted the importance of considering mixed-species infections when the quality of malaria diagnosis is assessed. As for other studies that extracted DNA from a comparably small volume of blood collected as DBS [14], it is important to remember that well-trained expert microscopists might reach a LOD similar or better than PCR. A calculation of the number of target molecules that are actually present in the aliquot that is used for the amplification reaction should be provided whenever an amplification protocol is presented. This includes a calculation of the corresponding volume of blood used as template for the reaction, considering dilutions made during extraction. Especially when a discussion of the limit of detection is presented, efforts should be made to quantify the proportion of genomes that are lost during extraction, for example by comparing densities determined by expert microscopy to target gene quantification by qPCR. Further, the number of copies per genome of the targeted gene needs to be reported. In this way, it will be possible to assess accurately the real limit of detection of any particular method. qPCR should not automatically be considered the most sensitive method to detect infections.

## Data Availability

Not applicable.
